# Identifying Host Genetic Risk Factors in the Context of Public Health Surveillance for Invasive Pneumococcal Disease

**DOI:** 10.1371/journal.pone.0023413

**Published:** 2011-08-15

**Authors:** Jairam R. Lingappa, Logan Dumitrescu, Shanta M. Zimmer, Ruth Lynfield, Janet M. McNicholl, Nancy E. Messonnier, Cynthia G. Whitney, Dana C. Crawford

**Affiliations:** 1 Departments of Global Health, Medicine and Pediatrics, University of Washington, Seattle, Washington, United States of America; 2 Center for Human Genetics Research, Vanderbilt University, Nashville, Tennessee, United States of America; 3 Department of Molecular Physiology and Biophysics, Vanderbilt University, Nashville, Tennessee, United States of America; 4 Emory University, Atlanta, Georgia, United States of America; 5 Minnesota Department of Health, St. Paul, Minnesota, United States of America; 6 Centers for Disease Control and Prevention, Atlanta, Georgia, United States of America; Leiden University Medical Center, The Netherlands

## Abstract

Host genetic factors that modify risk of pneumococcal disease may help target future public health interventions to individuals at highest risk of disease. We linked data from population-based surveillance for invasive pneumococcal disease (IPD) with state-based newborn dried bloodspot repositories to identify biological samples from individuals who developed invasive pneumococcal disease. Genomic DNA was extracted from 366 case and 732 anonymous control samples. TagSNPs were selected in 34 candidate genes thought to be associated with host response to invasive pneumococcal disease, and a total of 326 variants were successfully genotyped. Among 543 European Americans (EA) (182 cases and 361 controls), and 166 African Americans (AA) (53 cases and 113 controls), common variants in surfactant protein D (*SFTPD*) are consistently underrepresented in IPD. *SFTPD* variants with the strongest association for IPD are intronic rs17886286 (allelic OR 0.45, 95% confidence interval (CI) [0.25, 0.82], with p = 0.007) in EA and 5′ flanking rs12219080 (allelic OR 0.32, 95%CI [0.13, 0.78], with p = 0.009) in AA. Variants in *CD46* and *IL1R1* are also associated with IPD in both EA and AA, but with effects in different directions; *FAS*, *IL1B, IL4*, *IL10*, *IL12B*, *SFTPA1*, *SFTPB*, and *PTAFR* variants are associated (p≤0.05) with IPD in EA or AA. We conclude that variants in *SFTPD* may protect against IPD in EA and AA and genetic variation in other host response pathways may also contribute to risk of IPD. While our associations are not corrected for multiple comparisons and therefore must be replicated in additional cohorts, this pilot study underscores the feasibility of integrating public health surveillance with existing, prospectively collected, newborn dried blood spot repositories to identify host genetic factors associated with infectious diseases.

## Introduction

*Streptococcus pneumoniae* (pneumococcus) is a Gram-positive, encapsulated bacterium and a leading cause of pneumonia, meningitis and bloodstream infection in children and the elderly. An estimated 62,000 cases and 6,000 deaths occur annually from invasive pneumococcal disease (IPD) in the US.[1] Globally, IPD causes over a million deaths in children under the age of 5. Asymptomatic nasopharyngeal colonization with *S. pneumoniae* is widespread, but overall few of those colonized develop IPD. In the U.S., race-dependent infection rates are higher in African Americans and American Indians than in persons of European descent.[2]

In 2000 a protein-polysaccharide conjugate vaccine (PCV7) was licensed in the U.S. targeting seven of the 90 known serotypes of *S. pneumoniae*; in 2010 a 13-valent vaccine was also licensed. Population-based surveillance for IPD through the Active Bacterial Core surveillance (ABCs) in the US documented an 81% decline in incidence of IPD in children less than two years of age after introduction of PCV7.[1], [3] Nonetheless, cases of IPD caused by serotypes not covered by the vaccine continue to occur. Identification of sensitive and specific risk factors for IPD may be helpful in implementation of public health prevention of IPD.

Risk of IPD has been associated with pathogen virulence, host susceptibility and epidemiologic factors. Population-based public health surveillance for IPD has been a critical tool for identifying epidemiologic risk factors for disease including cigarette smoking, recent viral respiratory infection, chronic medical conditions, immunosuppression and lower socioeconomic status.[4] However, many of these factors are not easily amenable to public health prevention interventions.

Specific clinical conditions (e.g., HIV-1 infection, and sickle cell disease) and variants in some host genes [5] are known to modify risk for IPD. Furthermore, molecular pathways have been identified that may be critical in host response to IPD.[5] Public health surveillance for IPD, such as through ABCs, captures data for population-based case cohorts, but without prospective collection of host genetic samples. Most states separately implement population-based dried bloodspot collection from newborns (nDBS) for screening for specific inherited traits. Recently, we reported preliminary results of a pilot study to integrate ABCs invasive bacterial disease data with a state-based nDBS repository to identify genetic samples from cases and controls.[6] Here we use this approach to evaluate candidate host genetic variants as risk factors for IPD.

## Results

### tagSNPs associated with IPD

**Figure 1:**
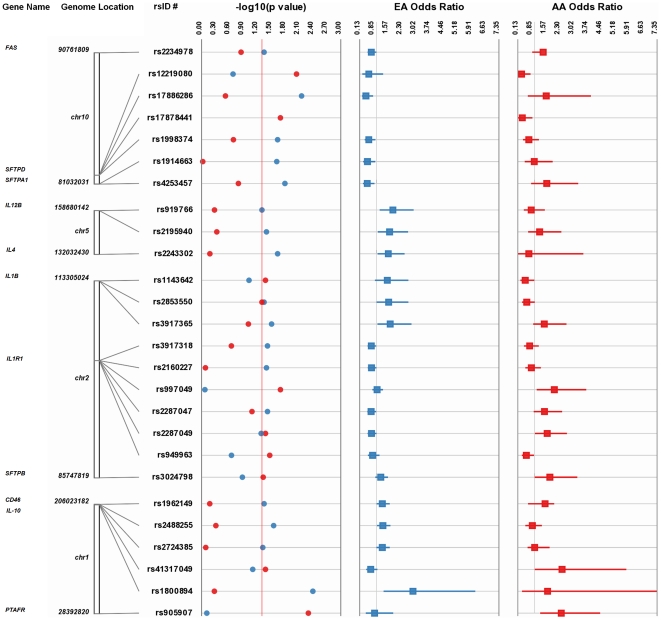
Summary of tagSNP associations for European-Americans and African Americans. Genomic and association characteristics for tagSNPs with p≤0.05 for IPD among either EA or AA are shown. Threshold lines for p = 0.05 (-log10 (0.05)  = 1.3) and OR = 1 are indicated in the relevant forest plots.

We performed single SNP allelic tests of association stratified by race/ethnicity in 543 European-Americans (EA), including 182 cases of IPD and 361 controls (Table 1) for 212 SNPs (Tables 2 and S1); and in 166 African-Americans (AA), including 53 cases and 113 controls for 287 SNPs. Because of the differences in sample size and its impact on power, we evaluated results based on both p-value and consistent direction of effect between the two ancestral groups. Overall, comparing IPD cases to controls, 17 tagSNPs in nine genes (*CD46*, *SFTPA1*, *SFTPD, IL1B*, *ILIR1*, *IL4*, *IL10*, *IL12B*, *FAS*) in EA and 11 tagSNPs in six genes (*CD46*, *SFTPB*, *SFTPD*, *IL1B, ILIR1*, and *PTAFR*) in AA have common variants that are associated at a liberal threshold of p≤0.05 (Tables 3 and S2, and Figure 1). Of these, three candidate genes, *SFTPD, CD46* and *IL1R1*, have tagSNPs associated with IPD in both EA and AA; these associations are further described below.

**Table 1:** Study Population Characteristics.

Characteristic	Surveillance cohort		Genotype cohort		Analysis cohort	
	Cases (N = 366)	Controls (N = 732)	Cases (N = 330)	Controls (N = 637)	Cases (N = 272)	Controls (N = 546)
**Demographics**						
Median Age in years (range)	1.11 (0-3.2)	1.11 (0-3.2)	1.12 (0-3.2)	1.11 (0-3.2)	1.11 (0-3.2)	1.09 (0-3.2)
Female^1^ # (%)	188 (51.4)	–	171 (51.8%)	–	144 (52.9%)	–
**Clinical classification # (%)**						
IPD	366 (100.0%)	–	330 (100.0%)	–	272 (100.0%)	–
Bacteremia only	245 (67.0%)	–	223 (67.6%)	–	182 (66.9%)	–
Pneumonia^2^	53 (14.2%)	–	43 (13.0%)	–	37 (13.6%)	–
Meningitis^2^	29 (8.2%)	–	28 (8.5%)	–	23 (8.5%)	–
Other^3^	39 (10.6%)	–	36 (11.0%)		30 (11.0%)	
Clinical Outcome – Death	5 (1.3%)		2 (0.6%)		2 (0.7%)	
**Race** 4						
European American	265 (72.2%)	534 (72.3%)	240 (72.7%)	452 (71.0%)	182 (66.9%)	361 (66.1%)
African American	62 (16.9%)	124 (16.8%)	53 (16.1%)	113 (17.7%)	53 (19.5%)	113 (20.7%)
American Indian	9 (2.45%)	18 (2.4%)	9 (2.7%)	17 (2.7%)	9 (3.3%)	17 (3.1%)
Asian	19 (5.18%)	38 (5.1%)	16 (4.8%)	31 (4.9%)	16 (5.9%)	31 (5.7%)
Other	12 (3.27%)	24 (3.2%)	12 (3.6%)	24 (3.8%)	12 (4.4%)	24 (4.4%)

1 Sex was identified for cases only.

2 With or without bacteremia.

3 Included in Other: sepsis (7), osteomyelitis or septic arthritis (6), sinusitis (2), otitis (18), cellulitis or abscess (5), UTI (1).

4 Race/ethnicity data based on chart abstracted medical records for cases and matched to newborn blood spot repository data for controls.

The Surveillance Cohort was based on all cases of IPD where an nDBS was identified. The Genotype Cohort was defined by samples with amplified genomic DNA of adequate quality for genotyping. The Analysis Cohort was defined by removing all outlier EA samples that STRUCTURE identified as having >10% African ancestry.

**Table 2:** Candidate Gene List.

	tagSNPs		
Candidate Gene (GeneID)	# Selected	# Genotyped	References
Innate Immune response and signaling pathway genes			
*CD46* (4179)	25	23	[6], [34], [35]
*CRP* (1401)	5	4	[36]-[38]
*FCGR2A* (2212)	6	3	[39], [40]
*FCGR3A* (2214)	9	1	[39], [40]
*IRAK4* (51135)	16	15	[41]
*LY96* [*MD-2*] (23643)	24	24	[42]
*MBL2* (4153)	10	10	[43], [44]
*MyD88* (4615)	3	2	[45]
*SFTPA1* (653509)	5	4	[46]
*SFTPB* (6439)	9	7	[47]
*SFTPD* (6441)	23	20	[46]
*TLR2* (7097)	11	10	[42], [48]
*TLR9* (54106)	7	3	[49]
**Pro- and anti-inflammatory mediators**			
*IFNG (3458)*	7	7	[50]
*IL1A (3552)*	13	12	[51], [52]
*IL1B (3553)*	19	13	[51]
*IL1R1 (3554)*	35	33	[53]
*IL4 (3565)*	18	15	[54]
*IL6 (3569)*	11	8	[55]
*IL8 (3576)*	3	3	[56], [57]
*IL10 (3586)*	7	7	[58]-[60]
*IL12A (3592)*	13	11	[61], [62]
*IL12B (3593)*	9	9	[61], [62]
*IL18 (3606)*	10	10	[63]-[65]
*LTA (4049)*	7	7	[66]
*TNFα (7124)*	5	4	[58], [67], [68]
**Coagulation pathway genes**			
*PROC* (5624)	14	5	[69], [70]
*PTAFR* (5724)	8	6	[71]
*SERPINE1* [PAI1] (5054)	9	9	[72], [73]
**Activation/apoptosis and immune regulatory genes**			
*CD40* (958)	11	10	[74]
*CD40LG* (959)	14	14	[74]
*CTLA4* (1493)	4	3	[75]
*FAS* (355)	7	7	[76]
*FASL* (356)	7	7	[76], [77]
**All 34 candidate genes**	**384**	**326**	

Candidate genes were selected based on the published literature and tagSNPs were selected using existing public variation databases.

**Table 3:** Summary of tagSNPs associated with IPD in European-Americans and/or African-Americans.

			European-American				African-American			
Gene	tagSNP	Gene Location	MAF (Cases)	MAF (Controls)	Allelic OR (95% CI)	p-value	MAF (Cases)	MAF (Controls)	Allelic OR (95% CI)	p-value
**Innate Immune response and signaling pathway genes**										
*CD46*	rs1962149	Intronic	0.48	0.42	**1.30 (1.01, 1.68)**	**0.045**	0.29	0.29	1.54 (0.66, 2.01)	0.667
	rs2488255	Intronic	0.49	0.42	**1.33 (1.03, 1.72)**	**0.028**	0.36	0.40	0.88 (0.52, 1.37)	0.492
	rs2724385	Intronic	0.53	0.47	**1.30 (1.00, 1.68)**	**0.048**	0.33	0.32	1.0 (0.64, 1.77)	0.813
	rs41317049	Intronic	0.10	0.14	0.70 (0.47, 1.04)	0.079	0.11	0.05	**2.42 (1.01, 5.75)**	**0.042**
*SFTPA1*	rs4253457	Intergenic	0.05	0.09	**0.52 (0.30, 0.89)**	**0.016**	0.15	0.10	1.63 (0.82, 3.26)	0.161
	rs1914663	Intronic	0.05	0.09	**0.53 (0.31, 0.93)**	**0.024**	0.14	0.14	0.98 (0.49, 1.93)	0.944
*SFTPB*	rs3024798	Intronic	0.40	0.36	1.22 (0.94, 1.59)	0.132	0.24	0.15	**1.79 (1.00, 3.21)**	**0.047**
*SFTPD*	rs1998374	Intronic	0.08	0.13	**0.60 (0.39, 0.94)**	**0.023**	0.22	0.28	0.70 (0.40, 1.22)	0.206
	rs12219080	Intronic	0.02	0.04	0.60 (0.27, 1.34)	0.210	0.06	0.16	**0.32 (0.13, 0.78)**	**0.009**
	rs17878441	Intronic	0.005	0.003	–	–	0.06	0.15	**0.36 (0.14, 0.88)**	**0.020**
	rs17886286	Intronic	0.04	0.08	**0.45 (0.25, 0.82)**	**0.007**	0.08	0.06	1.60 (0.65, 3.91)	0.306
**Pro- and anti-inflammatory mediators**										
*IL1B*	rs1143642	Intronic	0.07	0.05	1.56 (0.92, 2.65)	0.095	0.13	0.22	**0.51 (0.26, 0.98)**	**0.042**
	rs2853550	3' flanking	0.09	0.06	**1.63 (1.01, 2.65)**	**0.045**	0.23	0.33	**0.59 (0.35, 1.00)**	**0.050**
	rs3917365	3' flanking	0.09	0.05	**1.71 (1.05, 2.81)**	**0.031**	0.12	0.20	1.5 (0.93, 2.65)	0.098
*ILIR1*	rs949963	Intronic	0.13	0.16	0.8 (0.56, 1.15)	0.227	0.25	0.38	**0.57 (0.34, 0.96)**	**0.034**
	rs997049	Intronic	0.42	0.41	1.03 (0.79, 1.33)	0.848	0.24	0.14	**2.02 (1.11, 3.67)**	**0.020**
	rs2160227	Intronic	0.23	0.28	**0.74 (0.55, 0.99)**	**0.040**	0.46	0.51	0.83 (0.52, 1.32)	0.826
	rs2287047	Intronic	0.20	0.26	**0.72 (0.53, 0.98)**	**0.038**	0.57	0.46	1.51 (0.95, 2.42)	0.082
	rs2287049	Intronic	0.20	0.26	0.74 (0.54, 1.00)	0.052	0.48	0.36	**1.65 (1.02, 2.67)**	**0.042**
	rs3917318	3' untranslated	0.23	0.29	**0.73 (0.54, 0.98)**	**0.038**	0.31	0.38	0.74 (0.45, 1.21)	0.228
	rs3917272	Intronic	0	0	–	–	0.04	0	**8.75 (0.97, 79.22)**	**0.021**
*IL4*	rs2243302	5' flanking	0.13	0.08	**1.61 (1.06, 2.45)**	**0.023**	0.02	0.03	0.70 (1.39, 3.52)	0.663
*IL10*	rs1800894	5' flanking	0.05	0.02	**2.89 (1.37, 6.12)**	**0.004**	0.03	0.02	1.67 (0.35, 7.35)	0.530
*IL12B*	rs2195940	Intronic	0.11	0.07	**1.68 (1.07, 2.63)**	**0.022**	0.17	0.14	1.26 (0.67, 2.38)	0.470
	rs919766	Intronic	0.11	0.06	**1.85 (1.17, 2.92)**	**0.008**	0.17	0.20	0.82 (0.44, 1.52)	0.527
**Coagulation pathway genes**										
*PTAF*R	rs905907	Intergenic	0.03	0.03	0.90 (0.454, 1.86)	0.772	0.24	0.11	**2.38 (1.29, 4.39)**	**0.005**
**Activation/apoptosis and immune regulatory genes**										
*FAS*	rs2234978	Synonymous	0.20	0.26	**0.73 (0.54, 0.99)**	**0.045**	0.37	0.29	1.44 (0.88, 1.35)	0.141

Minor allele frequency (MAF), odds ratios (OR), 95% confidence intervals (CI), and p-values for SNPs associated with IPD at p≤0.05 in either European-Americans (n = 182 cases, 361 controls) or African-Americans (n = 53 cases, 113 controls) with allelic (2x2) association test. Associations with unadjusted p≤0.05 are in bold.

Variants in *SFTPD* have the strongest and most consistent associations across EA and AA. *SFTPD* intronic variants rs17886286 and rs1998374 are underrepresented among EA IPD cases compared to controls (allelic OR 0.45 and 0.60, with p = 0.007 and 0.023, respectively). These variants are in moderate linkage disequilibrium (LD) with each other in EA (SeattleSNPs r^2^ = 0.732) and thus do not represent independent associations. Also, these associations do not follow an additive genetic model given the observation that the genetic effect estimates (odds ratios) for heterozygotes and homozygotes are similar for each of these variants, individually (Table S2). Among AA, two different *SFTPD* variants (5′ flanking rs12219080 and intronic rs17878441) are underrepresented in IPD cases compared to controls (allelic OR 0.32, and 0.36, with p = 0.009 and 0.020, respectively). These SNPs are not in high LD in AA (SeattleSNPs r^2^ = 0.352). The association between *SFTPD* rs17878441 and IPD is specific to AA given that this SNP is very rare among the EA cohort presented here (1 heterozygote case and 1 heterozygote control among 508 individuals tested, overall MAF = 0.002) and monomorphic in reference populations such as CEU HapMap and SeattleSNPs EA. *SFTPD* rs1998374 and rs12219080 have consistent directions of effect in EA and AA, although neither is associated in both groups at p≤0.05 (Table 3 and Figure 1).

To explore the joint effects of associated variants in *SFTPD*, we calculated a weighted Genetic Risk Score (GRS) in EA and AA for significant associations with *SFTPD* variants (rs1998374 and rs17886286 in EA, and rs12219080 and rs17878441 in AA). Together, the two EA *SFTPD* variants were associated with IPD at an OR = 0.47 (95%CI: 0.26, 0.84) and p-value = 0.011. Additionally, the two AA *SFTPD* variants were associated with IPD at an OR = 0.36 (95%CI: 0.17, 0.77) and p-value = 0.009. This must be viewed cautiously, as the method assumes each SNP to be independently associated IPD status; however, the SNPs used in the GRS are in moderate LD (r^2^ = 0.50 in EA, r^2^ = 0.31 in AA).

Variants in *CD46* (three in EA and one in AA) are overrepresented in IPD compared to controls: among EA, intronic rs1962149, rs2488255, and rs2724385 have allelic ORs between 1.30 to 1.33, and p-values from 0.028 to 0.048 with moderate to strong LD (HapMap CEU r^2^ ranging from 0.684 to 1.00 in pair-wise comparisons). Intronic *CD46* rs41317049 is overrepresented in IPD among AA compared to controls (allelic OR 2.42 with p = 0.042); rs1962149 has a consistent direction of effect in EA and AA, but with p>0.05 among AA.

Finally, seven variants in *IL1R1* (three in EA and four in AA) are associated with IPD compared to controls at the p≤0.05 threshold. Among EA, intronic rs2160227, rs2287047, and rs3917318 are underrepresented among IPD cases compared to controls with very similar allelic ORs (0.72 to 0.73) and p-values (0.038–0.040). Among EA, LD is high for some (SeattleSNPs *r^2^* = 1.00 for rs2160227 and rs3917318, and r^2^ = 0.80 for rs2287047 and rs2287049), but not all variants (e.g., SeattleSNPs r^2^ = 0.302 for rs3917318 and rs2287047). The direction of effect for *IL1R1* variants associated in AA is not consistent: OR point estimates range from 0.57 to 8.75 with p-values of 0.020 to 0.042 (Table 3). Between EA and AA, the minor alleles for *IL1R1* intronic SNPs rs3917318 and rs2160227 and 5′ flanking rs949963 are consistently underrepresented among cases compared with control regardless of race/ethnicity.

Among genes with variants associated with IPD in EA but not AA, rs1800894 near *IL10* is most overrepresented in IPD with an allelic OR 2.89 and p = 0.004. Although not significant, the direction of effect is the same in AA for this *IL10* SNP (OR 1.67, 95% CI [0.35, 7.35]). *IL12B* intronic variants rs919766 and rs2195940 are overrepresented in EA for IPD (allelic OR 1.85, p = 0.008 and 1.68, p = 0.05, respectively); among EA, these variants are in complete LD (SeattleSNPs r^2^ = 1.00). Variant rs2195940 maintains the same direction of effect with a similar magnitude in AA, though this association does not reach statistical significance. Furthermore, in a genotypic test of association, this variant is associated with a large odds ratio among homozygotes compared to heterozygotes (Table S2). Finally, *SFTPA1* has two variants (intergenic rs4253457 and intronic rs1914663) with similar allelic OR in EA (0.52 and 0.53, respectively). Both are in complete LD in EA (SeattleSNPs; r^2^ = 1.00) but are in low LD in AA (SeattleSNPs r^2^ = 0.119). Of these, rs1914663 has a similar direction of effect among AA.

Among genes with variants associated with IPD in AA but not EA, rs905907 near *PTAFR* has the strongest association with an allelic OR of 2.38 and p = 0.005. The direction of effect in EA is opposite of that observed for AA for this association (OR  = 0.90; 95% CI: 0.454, 1.86).

Finally, for IL1B, variants rs2853550 and rs3917365 were overrepresented, but with low LD in EA (r^2^ = 0.352), while variants rs1143642 and rs2853550 were underrepresented and with low LD in AA (r^2^ = 0.141) but moderate LD in EA (r^2^ = 0.650). rs2853550 was the only tagSNP in our analysis that was associated (p≤0.05) in both AA and EA; but this SNP had opposite effects in each racial group.

### tagSNPs associated with specific clinical outcomes

As expected, most tests of association conducted within specific clinical outcomes give similar results as tests of association performed in the larger IPD study. However, we did observe potentially important differences. Specifically for pneumococcal bacteremia 15 tagSNPs in eight genes (*CD46*, *SFTPA1*, *SFTPD, ILIR1*, *IL4*, *IL12B*, *IL18*, and *MYD88*) and seven tagSNPs in seven genes (*MBL2*, *SFTPD, IL1B*, *ILIR1, IL4*, and *PTAFR*) are associated with cases of pneumococal bacteremia compared to controls in EA and AA, respectively (Table S3). Thus, in addition to the genes identified as having variants in EA associated with IPD, the *MYD88* variant rs7744 in the 3′ untranslated region is overrepresented in bacteremia cases among EA (allelic OR 1.49, p = 0.046) with a similar effect in AA (allelic OR 1.50), but which did not reach the p≤0.05 threshold. Also, the *MBL2* variant rs930507 is overrepresented in bacteremia cases among AA (allelic OR 1.82, p = 0.046) with a similar effect in EA (allelic OR 1.19) but with p>0.3.

Additional tagSNPs, particularly in *SFTPD*, are associated with pneumonia and meningitis syndromes in EA (Table S4). However, the sample sizes associated with these outcomes are small (n = 17 cases of meningitis, and n = 25 cases of pneumonia) precluding meaningful interpretation of these associations. The AA sample size associated with these outcomes is also too small (n = 2 cases and n = 6 cases, respectively) to permit further analysis.

## Discussion

In our evaluation of a population-based cohort for host genetic variation associated with IPD in children, we identified 27 tagSNPs in 11 genes (*CD46*, *SFTPA1*, *SFTPB*, *SFTPD, IL1B*, *IL1R1*, *IL4*, *IL10*, *IL12B*, FAS and *PTAFR*) associated in EA or AA at a liberal significance threshold of p≤0.05. In particular, in EA and AA, variants in the surfactant protein D (SP-D) encoded by *SFTPD* (gene ID 6441) are consistently underrepresented in IPD and pneumococcal bacteremia cases compared to controls, suggesting that variants in this gene or those in linkage disequilibrium may confer protection from IPD. This is the first study linking *SFTPD* gene variation specifically with clinical IPD.

SP-D is a member of the collectin subgroup in the C-type lectin superfamily including surfactant protein A (SP-A) and mannose binding protein. SP-D and SP-A are found primarily in the respiratory tract and other mucosal surfaces and recent data suggests that they impact respiratory infections on multiple levels. Surfactant collectins broadly bind carbohydrates and lipids on the surface of bacteria and viruses, with specific binding of SP-D to *S. pneumoniae* reported.[7] SP-D deficient (*sftpd^−/−^* knockout) mice are associated with persistent pneumococcal colonization, decreased clearance of bacterial pathogens, and early onset and increased levels of *S. pneumoniae* bacteremia in colonized mice.[7] Overall, collectins exhibit both pro- and anti-inflammatory effects[8]: SP-D stimulates phagocytosis and scavenging of apoptotic cells with pro-inflammatory consequences.[9] Yet, SP-D and SP-A bind SIRPα [10], TLR2, and TLR4,[11] and CD14[12] through their globular carbohydrate recognition domain (CRD) to down-regulate inflammatory cytokines; [11], [13] and *sftpd−/−* knockout mice exhibit high levels of pulmonary inflammation.[14] These findings have led to speculation that collectins have dual roles: if the collectin collaginase tail is bound in the absence of a pathogen stimulus, an anti-inflammatory response results possibly mitigating damage from incidental environmental stimuli.[15] However, when pathogen signals are present, pulmonary collectins may provide pro-inflammatory stimuli for pathogen phagocytosis and NF-κB-mediated cytokine release.[10] Further study is needed to confirm our tagSNP associations and further dissect how protection from IPD by *SFTPD* variants reflects regulatory functions of SP-D.

Our analysis also identified variants in other innate immune and coagulation pathway genes (e.g., *CRP* and *PTFAR*) and inflammatory mediators (e.g., *IL1R1*, *IL1B*, *IL12B* and *IL10*) that may be associated with IPD. Since, as an exploratory study, we did not correct for multiple comparisons, definitive interpretation of these findings will require confirmation in larger cohort studies. Nevertheless, our findings support multiple pathways being involved in host response to IPD. Recent studies also suggest that additional genes in the toll-like receptor-signaling pathway (e.g., *NFKB*, *IKB* and *MAL)* may influence response to IPD. [16]–[18] Furthermore, the collectin *MBL2* had variants overrepresented in pneumococcal bacteremia and meningitis, but not for overall IPD. This suggests the possibility of syndrome-specific host genetic associations, but our study was underpowered to definitively evaluate this.

For this analysis, we took an indirect association approach by selecting and genotyping SNPs that are either causative SNPs or in LD with the causative SNP. The latter situation most likely applies to the majority of SNPs found associated with IPD in this study, as 18 of the 27 (67%) associated SNPs are located in introns. Furthermore, of the four *SFTPD* variants associated with IPD in either EA and/or AA (including rs17886286 and rs1998374), all are intronic. Notably, in SeattleSNPs EA, rs17886286 and rs1998374 are in complete (r^2^ = 1.00) or high (r^2^ = 0.732) LD with rs3088308, a coding non-synonymous SNP, while in SeattleSNPs AA, these SNPs have little to no LD with rs3088308 (r^2^ = 0.002 for rs17886286 and r^2^ = 0.028 for rs1998374) and are not associated with IPD in AA. The two *SFTPD* variants that are associated with IPD in AA are in moderate LD with a different coding non-synonymous SNP, rs4469829, which is monomorphic in EA (r^2^ = 0.613 for rs17878441 and r^2^ = 0.620 for rs12219080). Furthermore, *SFTPD* variant rs721917, a non-synonymous SNP known to reduce serum levels of SP-D in EA, is in LD with rs1998374 in AA (SeattleSNPs r^2^ = 0.645) but not EA (SeattleSNPs r^2^ = 0.096). Thus, differences in LD patterns between ancestral populations may help to explain the disparate signals observed in EA compared to AA.

Our primary goal was to assess the feasibility of cross-linking surveillance data with an nDBS repository to perform tagSNP genomic studies, and toward this end we were highly successful: 82% of surveillance cases were linked to an nDBS, and 88% of samples successfully genotyped. Several key issues associated with this experience deserve emphasis. First, the completeness of IPD case surveillance in ABCs through use of active surveillance methods and routine audits of laboratory records combined with the overall low incidence of IPD in the general population support our assumption that controls were at low risk of having had IPD outside the surveillance time-period. Second, efficient linking of surveillance cases to nDBS samples was critical to minimize bias, but this linkage depends on consent requirements for nDBS use, which differ by state and continue to evolve.[19] Third, nearly a quarter of individuals identified as European-American through surveillance were found to have genetic characteristics indicating >10% African ancestry. Given differences in allele frequencies and LD between EA and AA populations, misclassification of ancestry can result in confounding. Although self-reported ancestry can be accurate in some settings,[20] our study underscores that more complete genetic evaluation for ancestry may be important to control for population stratification in some settings. Finally, the tagSNP approach we used is based on the assumption that most disease-causing variation will be captured through LD with common tagSNPs. Recent concern [21], [22] that low frequency host variation contributes substantial disease causation may underscore the need for large-scale gene sequencing and not a tagSNP analysis. Our earlier findings demonstrating that gene sequencing can be performed with high-fidelity using DNA from these nDBS samples [6] suggests that application of large-scale sequencing for initial variation discovery could be useful in future replication studies.

Our findings are not corrected for multiple comparisons and therefore should be viewed as preliminary with definitive proof requiring replication in additional cohorts. However, given our study results and the wealth of existing public health surveillance data and existing large repositories of nDBS, replication studies powered to detect associations with IPD and invasive disease caused by other vaccine preventable encapsulated bacteria (e.g., *N. meningitidis* and *H. influenzae*) should be feasible and could help further define host genetic risk factors for IPD and other infectious diseases and permit economic and attributable risk analyses to determine the usefulness of such risk factors for implementation of public health prevention interventions.

## Materials and Methods

### Ethics Statement

The protocol for this study was reviewed and approved by the institutional review boards at the Minnesota State Department of Health and the Centers for Disease Control and Prevention (CDC). Written informed consent was solicited from individuals providing nDBS or their guardians. The ethics approvals allowed for use of anonymous samples in cases where the individuals or their guardians provided written informed consent or where they could not be contacted; nDBS samples from individuals who declined consent were removed from this analysis. After determination of consent status, the database was made anonymous by delinking from surveillance identifiers, at which point CDC closed the project, allowing anonymous genotyping to proceed using the coded nDBS and study database. The University of Washington subsequently provided a certificate of exemption for this study.

### Subject Ascertainment

As previously described [6], IPD cases in the state of Minnesota were identified through Active Bacterial Core surveillance (ABCs), coordinated through the Centers for Disease Control and Prevention's Emerging Infections Program. Cases of IPD were defined as individuals born between January 1, 1997 and December 31, 2000 who had isolation of *S. pneumoniae* from a normally sterile site during the same time period. nDBS collected from cases during the course of normal newborn screening were identified by cross-linking ABCs identifiers with the Minnesota newborn screening program. For each case nDBS identified, two anonymous control nDBS were selected based on surveillance and newborn screening data by matching case and control race/ethnicity, date of birth, and, when possible, hospital of birth. Parents or guardians of cases were contacted by mail for written consent. Surveillance data and nDBS were included for all cases with parental consent and those who did not respond after two mailings. ABCs data and case and control nDBS were stripped of linkage to personal identifiers.

### Study cohort

Overall, 445 individuals were identified as meeting the IPD case definition. Of these cases, stored nDBS were identified for 366 (82%) with a median age of 1.1 years (range 0–3.2 years) and nearly equal numbers of males and females (Table 1). Anonymous nDBS were matched to cases by date of birth and identified race for 732 controls (82% of controls were also matched to cases by hospital of birth). Among these 1098 cases and controls (Surveillance Cohort), amplified DNA of adequate quality for genotyping was obtained from 967 (88%) representing 330 cases and 637 controls (Genotyped Cohort). Population substructure analysis indicated that 149 individuals (58 cases and 91 controls) in the Genotyped Cohort that had been specified in the surveillance data as having European American (EA) ancestry had genotypes consistent with ≥10% African ancestry. These cases were omitted from the final Analysis Cohort leaving 543 EA (182 cases and 361 controls), 166 AA (53 cases and 113 controls), and 109 from other ancestral backgrounds (Table 1). The 709 individuals (235 cases and 474 controls) of EA and AA race/ethnicity are referred to as the Analysis Cohort.

### Candidate Gene and tagSNP Selection

We selected 34 candidate genes (Tables 2 and S1) based on publications prior to 2007 reporting an association of the gene or protein product with IPD, pneumonia or sepsis, or laboratory evidence for a role in host response to *S. pneumoniae* or other encapsulated bacteria (e.g., *N. meningitidis*). TagSNPs were selected for African-Americans and European-Americans using software LDSelect [23] (r^2^>0.64; minor allele frequency >5%) and data from SeattleSNPs [24], the Environmental Genome Project [25], Perlegen [26], and the International HapMap Project [27]. Amplified DNA samples were genotyped for 384 tagSNPs with a custom Illumina GoldenGate assay [28] by the Center for Inherited Disease Research (CIDR) as part of the National Heart Lung and Blood Institute (NHLBI) Re-sequencing and Genotyping (RSnG) service.

### DNA Extraction and SNP Genotyping

De-identified genomic DNA extracted from 1,022 dried blood spots was whole genome amplified using a multiple displacement method (Molecular Staging, Inc. (MSI), New Haven, CT USA)[29] from a single 3-mm punch from ½ inch dried blood spots.

A total of 326 (85%) of 384 selected tagSNPs were successfully genotyped (Tables 2 and S1). Of 326 successfully genotyped, tagSNPs fell into 5 broad biological categories: 13 innate immune response genes with 126 SNPs (39%), 13 pro- and anti-inflammatory mediators with 139 SNPs (43%), three genes in coagulation pathways genes with 20 SNPs (6%), and five genes in activation/apoptosis pathways with 41 SNPs (12%) (Tables 2 and 1). Genes with 50% or more SNPs targeted but not successfully genotyped included: *FCGR3A* (8 of 9 SNPs failed genotyping), *PROC* (9 of 14 SNPs), *TLR9* (4 of 7 SNPs) and *FCGR2A* (3 of 6 SNPs). For the IPD analysis, a total of 212 (65%) and 287(88%) genotyped SNPs had >95% genotyping efficiency, had a minor allele frequency (MAF) >0.01, and met Hardy-Weinberg Equilibrium (HWE) criteria of p>0.0001 among EA and AA, respectively. The majority of SNPs genotyped for *CD40LG* (10 of 14) were out of HWE (p_EAcontrols_<0.0001) and subsequently omitted from further analyses. Genotyping associated with the bacteremia case definition included: 120 cases and 361 controls were included for EA with 213 SNPs passing QC, and 39 cases and 113 controls among AA with 281 SNPs passing QC. We also report genotyping data from EA for the 25 cases of pneumonia and 17 cases of meningitis compared to 361 controls, with 211 SNPs passing QC for each of those analyses.

### Study Population Demographics and Ancestry

Population stratification was evaluated using STRUCTURE (version 2.2, http://pritch.bsd.uchicago.edu/structure.html) prior to performing SNP quality control or testing for association.[30], [31] Discrepancies were noted between genotype inferred ancestry and surveillance database assigned ancestry using 274 SNPs with a minor allele frequency >1%. Samples labeled as European-American that were inferred to have >10% African-American ancestry were omitted from further analysis.

### Statistical Analysis

Only SNPs that passed quality control measures (minor allele frequency >1%, Hardy-Weinberg Equilibrium (HWE) p>0.0001 in controls, and genotyping efficiency >95%) were considered for analysis. All analyses were stratified by race/ethnicity, and tests of association were performed using PLINK [32] or STATA (version 10). Due to the small number of non-European, non-African samples, we did not analyze data from those samples. Exclusion of individual case or control samples due to genotyping quality control criteria and genetic ancestry disrupted the original case-control matching. In order to maximize the sample size for this exploratory analysis, tagSNP associations were assessed through an unmatched comparison of IPD cases to controls. Two-tailed χ^2^ tests were used with p≤0.05 considered significant without correction for multiple comparisons. Both allelic and genotypic tests of associations were performed for all SNPs. LD estimates were obtained through SeattleSNPs Genome Variation Server at http://gvs.gs.washington.edu/GVS/using data from the International HapMap Project [27] and SeattleSNPs [24] as indicated.

A weighted Genetic Risk Score (GRS) was calculated for *SFTPD* by PLINK for every participant, stratified by race/ethnicity, using SNPs that were associated with IPD status at p<0.05. The GRS is simply a sum across SNPs of the number of risk alleles (0, 1, or 2) at that SNP, multiplied by the odds ratio of the association. Participants with incomplete genotype data at any SNP used in the GRS were excluded from analysis. Logistic regression, with continuous GRS as the independent variable, was used to evaluate the joint effects of associated genetic variants with IPD status.

The primary analysis was based on ABCs data for IPD case status. We also evaluated other clinical outcomes of pneumococcal bacteremia, pneumonia, or meningitis, defined by *S. pneumoniae* cultured from blood only with no other site-specific diagnosis, isolation from pleural fluid or a clinician diagnosis of pneumonia, and isolation from cerebrospinal fluid or a clinician diagnosis of meningitis, respectively. Because of the small numbers of pneumonia and meningitis cases, we used Fisher's exact test for these analyses. Association results were also plotted graphically using Synthesis-View.[33]

## Supporting Information

Table S1 **Candidate SNP List.** Characteristics of tagging SNPs (based on linkage disequilibrium; r2) are listed for each selected gene and the genotyping outcome. The minor allele frequency (MAF) in the full genotyped cohort is also provided. SNPs with an asterisk (*) do not have rsIDs available. Flanking sequences used to design primers for these are listed below with the SNP indicated in brackets: TLR2_004667_rsNA:AGGTCCTGGATGCCCGAAGCTTCAAGAAAATACTGGTTGGGCACTTAGCTTTCCCTGTGGTTGCCAATCCCACGCAGGCC.GCCTCTAGCGTCTCGATTCG[C/T]TTTTCTCTGACCTGGAACCTCCGCCAAGCCCCCAGCTCTCTTCTTCGACCCAGCCTTGCACSGGGCAGCTGTCGGGGCAGGACCCGCCTCTGGCCTGTCG.TLR2_019165_rsNA:AATATTCATCTGAATCAAATTATCTATGATAACCTATTTAAATGAACAGCATCATGCACCTGAATTGGAGAAACAAAACTGAT.ATTTMAAATGATATAAA[C/T]TTAGTGTTAAATGTGGCCTCATGGATCAGACAGCTATCTGGTCCTTTCTGAGTCCATATAGCTGCCATTATTAAAAGTTCTGCACTCTATGACTCATCAC.TLR2_019827_rsNA: CAAAAGGAGAGATAATTAGACAGTTTGTAGACGAATTCATAACCCTAATTCTCTAATTGTTGGTAGCCTCCATTGATGATAAT.TAATAGTGGAGCCTTAG[A/G]TAAATACTACTATTGCTTCCCCGTTGATGAMCCCTGTAGCACACAGTGTCCCACAGGGAACTGTCTCTTGTGCCCCTCTAGCATATATTTTTATCTATGA.TLR2_023003_rsNA:ACTTAAACACTGCTTTTTGCATTTACTTCTAGGCAGGAAGACAGGGTTCTGATGGTGTGAGTCTCCTTCAACTCAGCAAACC. ACCTTGGTCTGCCTCGAG[C/T]TTCCAACACCCCTCCTGCCTGCTAGTGATAGGTGTGAGGCAGGTTGATGAACATGGAACTTTTTTCTTTTGGTCCCAAAGCATGCTACTCCTGGAGTTTC.
(DOC)

Table S2 **tagSNPs in candidate genes found associated with IPD.** For European-Americans (182 Cases and 361 Controls), and African-Americans (53 Cases and 113 Controls). Allelic (2×2) and genotypic (2×3) models are used to calculate allelic, heterozygote and homozygote OR, 95% confidence intervals (CI) and minor allele frequency (MAF). Variants are ordered by decreasing significance of the allelic p-value.
(DOC)

Table S3 **tagSNPs in candidate genes found associated with bacteremia.** For European-Americans (120 Cases and 361 Controls) with 213 SNPs passing QC (HWE >0.0001, MAF >0.01, Genotyping efficiency >90%) and African-Americans (39 Cases and 113 Controls) with 281 SNPs passing QC. Allelic (2×2) and genotypic (2×3) models are used to calculate allelic, heterozygote and homozygote OR, 95% confidence intervals (CI) and minor allele frequency (MAF). Variants are ordered by decreasing significance of the allelic p-value.
(DOC)

Table S4 **tagSNPs in candidate genes associated with pneumonia and meningitis for European-Americans (EA).** 25 Cases and 361 Controls with 211 SNPs passing QC (HWE >0.0001, MAF >0.01, Genotyping efficiency >90%). Allelic (2×2) and genotypic (2×3) models are used to calculate allelic, heterozygote and homozygote OR, 95% confidence intervals (CI) and minor allele frequency (MAF). Variants are ordered by decreasing significance of the allelic p-value.
(DOC)
