# Supplementary material for: Identifying Host Genetic Risk Factors in the Context of Public Health Surveillance for Invasive Pneumococcal Disease
Source: PLoS One. 2011 Aug 15;6(8):e23413. doi: 10.1371/journal.pone.0023413 (PMC3156135; doi:10.1371/journal.pone.0023413)
Supplement: Table S1 — Candidate SNP List. Characteristics of tagging SNPs (based on linkage disequilibrium; r2) are listed for each selected gene and the genotyping outcome. The minor allele frequency (MAF) in the full genotyped cohort is also provided. SNPs with an asterisk (*) do not have rsIDs available. Flanking sequences used to design primers for these are listed below with the SNP indicated in brackets: TLR2_004667_rsNA:AGGTCCTGGATGCCCGAAGCTTCAAGAAAATACTGGTTGGGCACTTAGCTTTCCCTGTGGTTGCCAATCCCACGCAGGCC.GCCTCTAGCGTCTCGATTCG[C/T]TTTTCTCTGACCTGGAACCTCCGCCAAGCCCCCAGCTCTCTTCTTCGACCCAGCCTTGCACSGGGCAGCTGTCGGGGCAGGACCCGCCTCTGGCCTGTCG.TLR2_019165_rsNA:AATATTCATCTGAATCAAATTATCTATGATAACCTATTTAAATGAACAGCATCATGCACCTGAATTGGAGAAACAAAACTGAT.ATTTMAAATGATATAAA[C/T]TTAGTGTTAAATGTGGCCTCATGGATCAGACAGCTATCTGGTCCTTTCTGAGTCCATATAGCTGCCATTATTAAAAGTTCTGCACTCTATGACTCATCAC.TLR2_019827_rsNA: CAAAAGGAGAGATAATTAGACAGTTTGTAGACGAATTCATAACCCTAATTCTCTAATTGTTGGTAGCCTCCATTGATGATAAT.TAATAGTGGAGCCTTAG[A/G]TAAATACTACTATTGCTTCCCCGTTGATGAMCCCTGTAGCACACAGTGTCCCACAGGGAACTGTCTCTTGTGCCCCTCTAGCATATATTTTTATCTATGA.TLR2_023003_rsNA:ACTTAAACACTGCTTTTTGCATTTACTTCTAGGCAGGAAGACAGGGTTCTGATGGTGTGAGTCTCCTTCAACTCAGCAAACC. ACCTTGGTCTGCCTCGAG[C/T]TTCCAACACCCCTCCTGCCTGCTAGTGATAGGTGTGAGGCAGGTTGATGAACATGGAACTTTTTTCTTTTGGTCCCAAAGCATGCTACTCCTGGAGTTTC. (DOC) [file pone.0023413.s001.doc]

**Table S1: Candidate SNP List**

| **Category** | **Gene name** | **SNP** | **Successfully genotyped** | **Chromosome** | **Genome position** | **Overall Minor Allele Frequency (MAF)** |
| --- | --- | --- | --- | --- | --- | --- |
| **Innate Immune response** | | | | | | |
|  | CD46 | rs11118514 | yes | 1 | 205990894 | 0.062238 |
|  | CD46 | rs11118516 | yes | 1 | 205995963 | 0.066107 |
|  | CD46 | rs17006738 | yes | 1 | 206002726 | 0.059896 |
|  | CD46 | rs17006830 | yes | 1 | 206007103 | 0.004136 |
|  | CD46 | rs1962149 | yes | 1 | 206023182 | 0.384615 |
|  | CD46 | rs1970530 | no | n/a | n/a | n/a |
|  | CD46 | rs2466572 | no | n/a | n/a | n/a |
|  | CD46 | rs2488255 | yes | 1 | 206008364 | 0.41242 |
|  | CD46 | rs2724384 | yes | 1 | 205996826 | 0.210921 |
|  | CD46 | rs2724385 | yes | 1 | 205998151 | 0.484273 |
|  | CD46 | rs2796267 | yes | 1 | 205991529 | 0.37931 |
|  | CD46 | rs2796269 | yes | 1 | 206003509 | 0.254727 |
|  | CD46 | rs35029161 | yes | 1 | 205993776 | 0.099682 |
|  | CD46 | rs35366573 | yes | 1 | 206025069 | 0.022013 |
|  | CD46 | rs41316827 | yes | 1 | 100000000 | 0.030761 |
|  | CD46 | rs41316835 | yes | 1 | 120000000 | 0.010858 |
|  | CD46 | rs41316841 | yes | 1 | 110000000 | 0 |
|  | CD46 | rs41317049 | yes | 1 | 90000000 | 0.123134 |
|  | CD46 | rs41317059 | yes | 1 | 40000000 | 0.014019 |
|  | CD46 | rs41317833 | yes | 1 | 30000000 | 0.002068 |
|  | CD46 | rs41317811 | yes | 1 | 50000000 | 0.00778 |
|  | CD46 | rs41318019 | yes | 1 | 80000000 | 0.069271 |
|  | CD46 | rs6664092 | yes | 1 | 206034363 | 0.006722 |
|  | CD46 | rs6671947 | yes | 1 | 206003114 | 0.099372 |
|  | CD46 | rs7545126 | yes | 1 | 206004859 | 0.116597 |
|  | CRP | rs1205 | yes | 1 | 157948857 | 0.315113 |
|  | CRP | rs1417938 | yes | 1 | 157950810 | 0.263845 |
|  | CRP | rs2808630 | no | n/a | n/a | n/a |
|  | CRP | rs3093058 | yes | 1 | 157951939 | 0.031574 |
|  | CRP | rs34200896 | yes | 1 | 157950216 | 0 |
|  | FCGR2A | rs10494360 | yes | 1 | 159742374 | 0.080922 |
|  | FCGR2A | rs17400517 | yes | 1 | 29282518 | 0.08447 |
|  | FCGR2A | rs1801274 | yes | 1 | 159746369 | 0.487138 |
|  | FCGR2A | rs382627 | no | n/a | n/a | n/a |
|  | FCGR2A | rs409763 | no | n/a | n/a | n/a |
|  | FCGR2A | rs430178 | no | n/a | n/a | n/a |
|  | FCGR3A | rs17410889 | yes | 1 | 159862370 | 0.017497 |
|  | FCGR3A | rs448740 | no | n/a | n/a | n/a |
|  | FCGR3A | rs1042206 | no | n/a | n/a | n/a |
|  | FCGR3A | rs15811 | no | n/a | n/a | n/a |
|  | FCGR3A | rs394260 | no | n/a | n/a | n/a |
|  | FCGR3A | rs396991 | no | n/a | n/a | n/a |
|  | FCGR3A | rs403016 | no | n/a | n/a | n/a |
|  | FCGR3A | rs428888 | no | n/a | n/a | n/a |
|  | FCGR3A | rs443082 | no | n/a | n/a | n/a |
|  | IRAK4 | rs1461567 | no | n/a | n/a | n/a |
|  | IRAK4 | rs4251429 | yes | 12 | 42956795 | 0.009307 |
|  | IRAK4 | rs4251439 | yes | 12 | 42960257 | 0.006722 |
|  | IRAK4 | rs4251460 | yes | 12 | 42965156 | 0.332278 |
|  | IRAK4 | rs4251467 | yes | 12 | 42966845 | 0.212042 |
|  | IRAK4 | rs4251473 | yes | 12 | 42968706 | 0.086618 |
|  | IRAK4 | rs4251475 | yes | 12 | 42969481 | 0.119689 |
|  | IRAK4 | rs4251483 | yes | 12 | 42971272 | 0.085084 |
|  | IRAK4 | rs4251494 | yes | 12 | 42973070 | 0.202004 |
|  | IRAK4 | rs4251513 | yes | 12 | 42976592 | 0.003661 |
|  | IRAK4 | rs4251514 | yes | 12 | 42976689 | 0.198834 |
|  | IRAK4 | rs4251520 | yes | 12 | 42977726 | 0.241579 |
|  | IRAK4 | rs4251522 | yes | 12 | 42977928 | 0.087859 |
|  | IRAK4 | rs4251535 | yes | 12 | 42981598 | 0.429957 |
|  | IRAK4 | rs4251545 | yes | 12 | 42982683 | 0.011435 |
|  | IRAK4 | rs4251551 | yes | 12 | 42983514 | 0 |
|  | LY96 | rs10088782 | yes | 8 | 75085896 | 0 |
|  | LY96 | rs10096996 | yes | 8 | 75075068 | 0.1125 |
|  | LY96 | rs10504554 | yes | 8 | 75082267 | 0.229787 |
|  | LY96 | rs10808798 | yes | 8 | 75069068 | 0.062565 |
|  | LY96 | rs11465996 | yes | 8 | 75064516 | 0.005688 |
|  | LY96 | rs11466002 | yes | 8 | 75084989 | 0.017188 |
|  | LY96 | rs11466004 | yes | 8 | 75103829 | 0.183442 |
|  | LY96 | rs11783456 | yes | 8 | 75080725 | 0.411672 |
|  | LY96 | rs11786591 | yes | 8 | 75078898 | 0.004145 |
|  | LY96 | rs1426060 | yes | 8 | 75081425 | 0.007261 |
|  | LY96 | rs16938755 | yes | 8 | 75066690 | 0.114062 |
|  | LY96 | rs16938760 | yes | 8 | 75082892 | 0.039583 |
|  | LY96 | rs16938761 | yes | 8 | 75083152 | 0.195606 |
|  | LY96 | rs17226566 | yes | 8 | 75082466 | 0.233684 |
|  | LY96 | rs17226734 | yes | 8 | 75087565 | 0.003119 |
|  | LY96 | rs17324476 | yes | 8 | 75088836 | 0 |
|  | LY96 | rs1812615 | yes | 8 | 75064082 | 0.442166 |
|  | LY96 | rs1991262 | yes | 8 | 75081883 | 0.011893 |
|  | LY96 | rs2114169 | yes | 8 | 75103440 | 0.023908 |
|  | LY96 | rs4737403 | yes | 8 | 75104088 | 0.013598 |
|  | LY96 | rs4738414 | yes | 8 | 75090646 | 0 |
|  | LY96 | rs6472812 | yes | 8 | 75079638 | 0.105729 |
|  | LY96 | rs7820086 | yes | 8 | 75103065 | 0 |
|  | LY96 | rs7822407 | yes | 8 | 75070367 | 0.084808 |
|  | MBL2 | rs10824793 | yes | 10 | 54199486 | 0.324973 |
|  | MBL2 | rs11003125 | yes | 10 | 54202020 | 0.005694 |
|  | MBL2 | rs1800451 | yes | 10 | 54201232 | 0.410011 |
|  | MBL2 | rs1838065 | yes | 10 | 54199263 | 0.468584 |
|  | MBL2 | rs2099903 | yes | 10 | 54195863 | 0.043113 |
|  | MBL2 | rs2165813 | yes | 10 | 54195990 | 0.023983 |
|  | MBL2 | rs7095891 | yes | 10 | 54201467 | 0.16828 |
|  | MBL2 | rs7096206 | yes | 10 | 54201691 | 0.204857 |
|  | MBL2 | rs930507 | yes | 10 | 54198272 | 0.034139 |
|  | MBL2 | rs930509 | yes | 10 | 54198359 | 0.004663 |
|  | MYD88 | rs4988457 | no | n/a | n/a | n/a |
|  | MYD88 | rs6853 | yes | 3 | 38159374 | 0.456216 |
|  | MYD88 | rs7744 | yes | 3 | 38159025 | 0.156748 |
|  | SFTPA1 | rs1346418 | yes | 10 | 81352234 | 0.035115 |
|  | SFTPA1 | rs1427815 | no | n/a | n/a | n/a |
|  | SFTPA1 | rs1914663 | yes | 10 | 81361905 | 0.025574 |
|  | SFTPA1 | rs4253457 | yes | 10 | 10052892 | 0.355249 |
|  | SFTPA1 | rs4253505 | yes | 10 | 49056675 | 0.145723 |
|  | SFTPB | rs1130866 | yes | 2 | 85747252 | 0.018097 |
|  | SFTPB | rs2040349 | no | n/a | n/a | n/a |
|  | SFTPB | rs3024791 | no | n/a | n/a | n/a |
|  | SFTPB | rs3024798 | yes | 2 | 85747819 | 0.081924 |
|  | SFTPB | rs3024802 | yes | 2 | 85745671 | 0.01782 |
|  | SFTPB | rs3024811 | yes | 2 | 85743659 | 0 |
|  | SFTPB | rs3024822 | yes | 2 | 85740101 | 0.124347 |
|  | SFTPB | rs3024832 | yes | 2 | 85742295 | 0 |
|  | SFTPB | rs7316 | yes | 2 | 85739524 | 0.14377 |
|  | SFTPD | rs1570328 | yes | 10 | 108588058 | 0.120635 |
|  | SFTPD | rs17885087 | no | n/a | n/a | n/a |
|  | SFTPD | rs17886252 | yes | 10 | 26799140 | 0.433754 |
|  | SFTPD | rs1923537 | yes | 10 | 81685188 | 0.122268 |
|  | SFTPD | rs1923541 | yes | 10 | 81680275 | 0.337222 |
|  | SFTPD | rs2245545 | yes | 10 | 81684739 | 0.010384 |
|  | SFTPD | rs2758554 | yes | 10 | 81681060 | 0.01298 |
|  | SFTPD | rs12219080 | yes | 10 | 81699259 | 0.020186 |
|  | SFTPD | rs12776624 | yes | 10 | 81690873 | 0.075026 |
|  | SFTPD | rs17878441 | yes | 10 | 81693206 | 0.053702 |
|  | SFTPD | rs17884887 | yes | 10 | 81693413 | 0.359391 |
|  | SFTPD | rs17885228 | no | n/a | n/a | n/a |
|  | SFTPD | rs17885295 | no | n/a | n/a | n/a |
|  | SFTPD | rs17886233 | yes | 10 | 81686809 | 0.004149 |
|  | SFTPD | rs17886286 | yes | 10 | 81693832 | 0 |
|  | SFTPD | rs17886630 | yes | 10 | 81689289 | 0.141361 |
|  | SFTPD | rs1998374 | yes | 10 | 81692702 | 0.346318 |
|  | SFTPD | rs2181204 | yes | 10 | 81694492 | 0.033333 |
|  | SFTPD | rs2758545 | yes | 10 | 81690070 | 0.061458 |
|  | SFTPD | rs2819097 | yes | 10 | 81689961 | 0.074324 |
|  | SFTPD | rs4469829 | yes | 10 | 81687791 | 0.494192 |
|  | SFTPD | rs7078012 | yes | 10 | 81695413 | 0 |
|  | SFTPD | rs721917 | yes | 10 | 81696304 | 0.054574 |
|  | TLR2 | rs11938228 | yes | 4 | 154841396 | 0.266527 |
|  | TLR2 | rs2083287 | yes | 4 | 154827779 | 0.014004 |
|  | TLR2 | rs3804099 | yes | 4 | 154844106 | 0.165781 |
|  | TLR2 | rs3804100 | yes | 4 | 154844859 | 0.330499 |
|  | TLR2 | rs4235232 | yes | 4 | 154837534 | 0.040933 |
|  | TLR2 | rs4696483 | yes | 4 | 154838705 | 0.437286 |
|  | TLR2 | rs5743708 | yes | 4 | 154845767 | 0.470936 |
|  | TLR2 | TLR2_004667_rsNA* | yes | 4 | 60000000 | 0.27 |
|  | TLR2 | TLR2_019165_rsNA* | no | n/a | n/a | n/a |
|  | TLR2 | TLR2_019827_rsNA* | yes | 4 | 10000000 | 0.257368 |
|  | TLR2 | TLR2_023003_rsNA* | yes | 4 | 20000000 | 0.190011 |
|  | TLR9 | rs17846009 | no | n/a | n/a | n/a |
|  | TLR9 | rs187084 | no | n/a | n/a | n/a |
|  | TLR9 | rs5743838 | yes | 3 | 52239910 | 0.306349 |
|  | TLR9 | rs5743841 | yes | 3 | 52239430 | 0.471698 |
|  | TLR9 | rs5743844 | yes | 3 | 52237348 | 0.150785 |
|  | TLR9 | rs5743846 | no | n/a | n/a | n/a |
|  | TLR9 | rs5743849 | no | n/a | n/a | n/a |
| **Pro/Anti Inflammatory** | | | | | | |
|  | IFNG | rs1861493 | yes | 12 | 68215247 | 0.402583 |
|  | IFNG | rs1861494 | yes | 12 | 68215460 | 0.143085 |
|  | IFNG | rs2069707 | yes | 12 | 68218336 | 0.418605 |
|  | IFNG | rs2069714 | yes | 12 | 68215095 | 0.283133 |
|  | IFNG | rs2069725 | yes | 12 | 68212436 | 0 |
|  | IFNG | rs2069727 | yes | 12 | 68212273 | 0.023784 |
|  | IFNG | rs2069728 | yes | 12 | 68211834 | 0.428243 |
|  | IL1A | rs1533463 | no | n/a | n/a | n/a |
|  | IL1A | rs17561 | yes | 2 | 113253694 | 0.149104 |
|  | IL1A | rs1878318 | yes | 2 | 113261656 | 0.104478 |
|  | IL1A | rs1878321 | yes | 2 | 113260905 | 0.007277 |
|  | IL1A | rs2071375 | yes | 2 | 113251909 | 0.047996 |
|  | IL1A | rs2856841 | yes | 2 | 113253810 | 0.102094 |
|  | IL1A | rs3783516 | yes | 2 | 113262149 | 0.45301 |
|  | IL1A | rs3783525 | yes | 2 | 113258290 | 0.005214 |
|  | IL1A | rs3783546 | yes | 2 | 113251301 | 0.28135 |
|  | IL1A | rs3783564 | yes | 2 | 113262846 | 0.14482 |
|  | IL1A | rs3783565 | yes | 2 | 113262762 | 0.440866 |
|  | IL1A | rs3783590 | yes | 2 | 113248196 | 0.101387 |
|  | IL1A | rs3783591 | yes | 2 | 113247985 | 0.417373 |
|  | IL1B | rs3917366 | yes | 2 | 113300351 | 0.408659 |
|  | IL1B | rs3917368 | yes | 2 | 65965962 | 0.135504 |
|  | IL1B | rs3917372 | yes | 2 | 546832 | 0.248152 |
|  | IL1B | rs3917377 | no | n/a | n/a | n/a |
|  | IL1B | rs1143627 | no | n/a | n/a | n/a |
|  | IL1B | rs1143629 | no | n/a | n/a | n/a |
|  | IL1B | rs1143630 | yes | 2 | 113308126 | 0.064331 |
|  | IL1B | rs1143634 | yes | 2 | 113306861 | 0.009886 |
|  | IL1B | rs1143637 | yes | 2 | 113305804 | 0.140761 |
|  | IL1B | rs1143639 | no | n/a | n/a | n/a |
|  | IL1B | rs1143642 | yes | 2 | 113305024 | 0.011942 |
|  | IL1B | rs16944 | no | n/a | n/a | n/a |
|  | IL1B | rs2853550 | yes | 2 | 113303592 | 0.240191 |
|  | IL1B | rs3136558 | yes | 2 | 113307746 | 0.092437 |
|  | IL1B | rs3917348 | yes | 2 | 113310219 | 0.068134 |
|  | IL1B | rs3917356 | yes | 2 | 113308834 | 0.008377 |
|  | IL1B | rs3917360 | yes | 2 | 113306222 | 0.044211 |
|  | IL1B | rs3917362 | no | n/a | n/a | n/a |
|  | IL1B | rs3917365 | yes | 2 | 113302940 | 0.36859 |
|  | IL1R1 | rs2110726 | no | n/a | n/a | n/a |
|  | IL1R1 | rs2160227 | yes | 2 | 102149787 | 0.022396 |
|  | IL1R1 | rs2192752 | yes | 2 | 102135805 | 0.409478 |
|  | IL1R1 | rs2228139 | yes | 2 | 102148081 | 0.340336 |
|  | IL1R1 | rs2287047 | yes | 2 | 102140486 | 0.056139 |
|  | IL1R1 | rs2287049 | yes | 2 | 102137170 | 0.386268 |
|  | IL1R1 | rs3732131 | yes | 2 | 102161035 | 0.02453 |
|  | IL1R1 | rs3917225 | yes | 2 | 102135734 | 0.178534 |
|  | IL1R1 | rs3917229 | yes | 2 | 102137010 | 0.007246 |
|  | IL1R1 | rs3917236 | yes | 2 | 102138700 | 0.40981 |
|  | IL1R1 | rs3917242 | yes | 2 | 102140700 | 0.020186 |
|  | IL1R1 | rs3917243 | yes | 2 | 102141420 | 0.493651 |
|  | IL1R1 | rs3917249 | yes | 2 | 102141882 | 0.039657 |
|  | IL1R1 | rs3917254 | yes | 2 | 102142950 | 0.010417 |
|  | IL1R1 | rs3917267 | no | n/a | n/a | n/a |
|  | IL1R1 | rs3917272 | yes | 2 | 102146052 | 0.077325 |
|  | IL1R1 | rs3917273 | yes | 2 | 102146151 | 0.025389 |
|  | IL1R1 | rs3917281 | yes | 2 | 102147085 | 0.154088 |
|  | IL1R1 | rs3917285 | yes | 2 | 102147598 | 0.008273 |
|  | IL1R1 | rs3917289 | yes | 2 | 102148343 | 0.032105 |
|  | IL1R1 | rs3917292 | yes | 2 | 102149484 | 0.06978 |
|  | IL1R1 | rs3917300 | yes | 2 | 102152956 | 0.157143 |
|  | IL1R1 | rs3917306 | yes | 2 | 102155271 | 0.074273 |
|  | IL1R1 | rs3917308 | yes | 2 | 102156130 | 0.014048 |
|  | IL1R1 | rs3917318 | yes | 2 | 102159192 | 0.029787 |
|  | IL1R1 | rs3917320 | yes | 2 | 102159307 | 0.030335 |
|  | IL1R1 | rs3917327 | yes | 2 | 102160811 | 0.246855 |
|  | IL1R1 | rs3917332 | yes | 2 | 102162956 | 0.005705 |
|  | IL1R1 | rs3917333 | yes | 2 | 102163215 | 0.02228 |
|  | IL1R1 | rs3917335 | yes | 2 | 102137725 | 0.247634 |
|  | IL1R1 | rs3917342 | yes | 2 | 102151803 | 0.050328 |
|  | IL1R1 | rs871658 | yes | 2 | 102138297 | 0.40981 |
|  | IL1R1 | rs949963 | yes | 2 | 102136218 | 0.02183 |
|  | IL1R1 | rs951193 | yes | 2 | 102152231 | 0.118546 |
|  | IL1R1 | rs997049 | yes | 2 | 102148865 | 0.43617 |
|  | IL4 | rs2243202 | yes | 5 | 132027282 | 0.043503 |
|  | IL4 | rs2243206 | no | n/a | n/a | n/a |
|  | IL4 | rs2243208 | no | n/a | n/a | n/a |
|  | IL4 | rs2243218 | yes | 5 | 132029923 | 0 |
|  | IL4 | rs2243219 | yes | 5 | 132030024 | 0.186896 |
|  | IL4 | rs2243297 | no | n/a | n/a | n/a |
|  | IL4 | rs2243302 | yes | 5 | 33485610 | 0.337953 |
|  | IL4 | rs2070874 | yes | 5 | 132037609 | 0.10343 |
|  | IL4 | rs2227284 | yes | 5 | 132040624 | 0.402793 |
|  | IL4 | rs2243235 | yes | 5 | 132034657 | 0.090104 |
|  | IL4 | rs2243246 | yes | 5 | 132036307 | 0.059709 |
|  | IL4 | rs2243247 | yes | 5 | 132036505 | 0.014301 |
|  | IL4 | rs2243251 | yes | 5 | 132037686 | 0.431277 |
|  | IL4 | rs2243252 | yes | 5 | 132038487 | 0.252621 |
|  | IL4 | rs2243253 | yes | 5 | 132038660 | 0.086589 |
|  | IL4 | rs2243263 | yes | 5 | 132041198 | 0.376963 |
|  | IL4 | rs2243274 | yes | 5 | 132042731 | 0.053571 |
|  | IL4 | rs2243283 | yes | 5 | 132044492 | 0.019792 |
|  | IL6 | rs2069840 | no | n/a | n/a | n/a |
|  | IL6 | rs1800797 | yes | 7 | 22817330 | 0.356462 |
|  | IL6 | rs2066992 | yes | 7 | 22819358 | 0.002588 |
|  | IL6 | rs2069824 | no | n/a | n/a | n/a |
|  | IL6 | rs2069827 | yes | 7 | 22816565 | 0.015057 |
|  | IL6 | rs2069838 | no | n/a | n/a | n/a |
|  | IL6 | rs2069842 | yes | 7 | 22820419 | 0.184375 |
|  | IL6 | rs2069845 | yes | 7 | 22821258 | 0.100727 |
|  | IL6 | rs2069849 | yes | 7 | 22822265 | 0.16195 |
|  | IL6 | rs2069855 | yes | 7 | 22823734 | 0.015042 |
|  | IL6 | rs2069861 | yes | 7 | 22822763 | 0.247126 |
|  | IL8 | rs2227532 | yes | 4 | 74824396 | 0.007299 |
|  | IL8 | rs2227538 | yes | 4 | 74825239 | 0.171792 |
|  | IL8 | rs4073 | yes | 4 | 74824888 | 0.232143 |
|  | IL10 | rs1554286 | yes | 1 | 205010856 | 0.251613 |
|  | IL10 | rs1800894 | yes | 1 | 205013289 | 0.023316 |
|  | IL10 | rs1878672 | yes | 1 | 205010336 | 0.39837 |
|  | IL10 | rs3021094 | yes | 1 | 205011575 | 0.105291 |
|  | IL10 | rs3024493 | yes | 1 | 205010591 | 0.107781 |
|  | IL10 | rs3024494 | yes | 1 | 205009974 | 0.013062 |
|  | IL10 | rs3024498 | yes | 1 | 205008152 | 0.211928 |
|  | IL12A | rs2243148 | yes | 3 | 64287132 | 0.27577 |
|  | IL12A | rs2243149 | yes | 3 | 161198406 | 0.047297 |
|  | IL12A | rs2243154 | yes | 3 | 161198936 | 0.040146 |
|  | IL12A | rs668998 | yes | 3 | 161198245 | 0.016029 |
|  | IL12A | rs2227314 | no | n/a | n/a | n/a |
|  | IL12A | rs2243113 | yes | 3 | 161188557 | 0.02588 |
|  | IL12A | rs2243115 | yes | 3 | 161188974 | 0.309674 |
|  | IL12A | rs2243128 | yes | 3 | 161193205 | 0.22572 |
|  | IL12A | rs2243132 | yes | 3 | 161194924 | 0.203684 |
|  | IL12A | rs2243135 | yes | 3 | 161195687 | 0.257173 |
|  | IL12A | rs475825 | yes | 3 | 161193022 | 0.322072 |
|  | IL12A | rs582054 | no | n/a | n/a | n/a |
|  | IL12A | rs583911 | yes | 3 | 161193084 | 0.295983 |
|  | IL12B | rs1368439 | yes | 5 | 158674592 | 0.152688 |
|  | IL12B | rs2195940 | yes | 5 | 158676930 | 0.315761 |
|  | IL12B | rs2569253 | yes | 5 | 158683571 | 0.050679 |
|  | IL12B | rs2569254 | yes | 5 | 158683827 | 0.069755 |
|  | IL12B | rs2853694 | yes | 5 | 158681666 | 0.028481 |
|  | IL12B | rs3212217 | yes | 5 | 158687708 | 0.177282 |
|  | IL12B | rs3213103 | yes | 5 | 158379014 | 0.039071 |
|  | IL12B | rs3213105 | yes | 5 | 158678277 | 0.307858 |
|  | IL12B | rs919766 | yes | 5 | 158680142 | 0.169189 |
|  | IL18 | rs1946519 | yes | 11 | 111540717 | 0.001551 |
|  | IL18 | rs360722 | yes | 11 | 111531913 | 0.305408 |
|  | IL18 | rs3882891 | yes | 11 | 111519971 | 0.031646 |
|  | IL18 | rs5744229 | yes | 11 | 111539787 | 0.361905 |
|  | IL18 | rs5744247 | yes | 11 | 111531366 | 0.328947 |
|  | IL18 | rs5744258 | yes | 11 | 111526977 | 0.256586 |
|  | IL18 | rs5744266 | yes | 11 | 111523735 | 0.256329 |
|  | IL18 | rs5744280 | yes | 11 | 111521724 | 0.261955 |
|  | IL18 | rs5744283 | yes | 11 | 111521210 | 0.320864 |
|  | IL18 | rs795467 | yes | 11 | 111536290 | 0.268008 |
|  | LTA | rs1800629 | yes | 6 | 33141246 | 0.055381 |
|  | LTA | rs1800750 | yes | 6 | 33141178 | 0.018172 |
|  | LTA | rs2229094 | yes | 6 | 33138771 | 0.020249 |
|  | LTA | rs2239704 | yes | 6 | 33138356 | 0.46266 |
|  | LTA | rs2844482 | yes | 6 | 33137982 | 0.156017 |
|  | LTA | rs361525 | yes | 6 | 33141316 | 0.005171 |
|  | LTA | rs909253 | yes | 6 | 33138528 | 0.07241 |
|  | TNF | rs1800610 | yes | 6 | 33142042 | 0.241885 |
|  | TNF | rs2228088 | yes | 6 | 33141820 | 0.001035 |
|  | TNF | rs3093662 | yes | 6 | 33142404 | 0 |
|  | TNF | rs4645843 | yes | 6 | 33142777 | 0.448663 |
|  | TNF | rs769178 | no | n/a | n/a | n/a |
| **Coagulation pathways** | | | | | | |
|  | PROC | rs1518759 | no | n/a | n/a | n/a |
|  | PROC | rs1799810 | yes | 2 | 127892510 | 0.116279 |
|  | PROC | rs2069897 | yes | 2 | 127890624 | 0.317842 |
|  | PROC | rs2069901 | no | n/a | n/a | n/a |
|  | PROC | rs2069906 | no | n/a | n/a | n/a |
|  | PROC | rs2069910 | no | n/a | n/a | n/a |
|  | PROC | rs2069912 | no | n/a | n/a | n/a |
|  | PROC | rs2069915 | no | n/a | n/a | n/a |
|  | PROC | rs2069918 | no | n/a | n/a | n/a |
|  | PROC | rs2069926 | yes | 2 | 127899815 | 0.01147 |
|  | PROC | rs2069927 | yes | 2 | 127900225 | 0.407267 |
|  | PROC | rs2069928 | no | n/a | n/a | n/a |
|  | PROC | rs2069932 | no | n/a | n/a | n/a |
|  | PROC | rs5937 | yes | 2 | 127901240 | 0.046025 |
|  | PTAFR | rs2481974 | no | n/a | n/a | n/a |
|  | PTAFR | rs905907 | yes | 1 | 28392820 | 0.056545 |
|  | PTAFR | rs17162976 | yes | 1 | 28370650 | 0.020228 |
|  | PTAFR | rs17162977 | yes | 1 | 28371468 | 0.008299 |
|  | PTAFR | rs17162980 | yes | 1 | 28376190 | 0.008808 |
|  | PTAFR | rs313990 | yes | 1 | 28362279 | 0.251596 |
|  | PTAFR | rs5938 | no | n/a | n/a | n/a |
|  | PTAFR | rs5939 | yes | 1 | 28349107 | 0.009989 |
|  | SERPINE1 | rs11178 | yes | 7 | 100567804 | 0.011905 |
|  | SERPINE1 | rs2227631 | yes | 7 | 100556258 | 0.394709 |
|  | SERPINE1 | rs2227636 | yes | 7 | 100557337 | 0.062369 |
|  | SERPINE1 | rs2227657 | yes | 7 | 100560179 | 0.177895 |
|  | SERPINE1 | rs2227667 | yes | 7 | 100561469 | 0.057173 |
|  | SERPINE1 | rs2227692 | yes | 7 | 100565964 | 0.089342 |
|  | SERPINE1 | rs2227693 | yes | 7 | 100566044 | 0.028986 |
|  | SERPINE1 | rs2227705 | yes | 7 | 100569712 | 0.004688 |
|  | SERPINE1 | rs6090 | yes | 7 | 100558443 | 0.005705 |
| **Activation/apoptosis and immune regulatory genes** | | | | | | |
|  | CD40 | rs11086998 | yes | 20 | 44190931 | 0.263018 |
|  | CD40 | rs11569314 | yes | 20 | 44183241 | 0.022349 |
|  | CD40 | rs11569315 | yes | 20 | 44183831 | 0.237261 |
|  | CD40 | rs11569321 | no | n/a | n/a | n/a |
|  | CD40 | rs1535045 | yes | 20 | 44181506 | 0.008827 |
|  | CD40 | rs17177493 | yes | 20 | 44184382 | 0.134146 |
|  | CD40 | rs2092566 | yes | 20 | 44183321 | 0.126874 |
|  | CD40 | rs3746821 | yes | 20 | 44188518 | 0.017296 |
|  | CD40 | rs3765459 | yes | 20 | 44190814 | 0.196986 |
|  | CD40 | rs6104478 | yes | 20 | 44189521 | 0.435884 |
|  | CD40 | rs7273698 | yes | 20 | 44190230 | 0.029749 |
|  | CD40LG | rs1126535 | yes | 23 | 136094231 | 0.177971 |
|  | CD40LG | rs3092922 | yes | 23 | 136105232 | 0 |
|  | CD40LG | rs3092923 | yes | 23 | 136104861 | 0.193953 |
|  | CD40LG | rs3092924 | yes | 23 | 136104122 | 0.056657 |
|  | CD40LG | rs3092925 | yes | 23 | 136103958 | 0.05344 |
|  | CD40LG | rs3092926 | yes | 23 | 136103348 | 0.054207 |
|  | CD40LG | rs3092928 | yes | 23 | 136102938 | 0.053349 |
|  | CD40LG | rs3092929 | yes | 23 | 136102599 | 0.158648 |
|  | CD40LG | rs3092931 | yes | 23 | 136101389 | 0.002254 |
|  | CD40LG | rs3092934 | yes | 23 | 136100783 | 0.000565 |
|  | CD40LG | rs3092935 | yes | 23 | 136100646 | 0.01875 |
|  | CD40LG | rs3092936 | yes | 23 | 136099881 | 0.089357 |
|  | CD40LG | rs3092937 | yes | 23 | 136099037 | 0.001128 |
|  | CD40LG | rs3092938 | yes | 23 | 136098168 | 0.055399 |
|  | CTLA4 | rs231775 | yes | 2 | 204440959 | 0.059039 |
|  | CTLA4 | rs231777 | yes | 2 | 204441833 | 0.009834 |
|  | CTLA4 | rs231778 | no | n/a | n/a | n/a |
|  | CTLA4 | rs231779 | yes | 2 | 204442732 | 0.486037 |
|  | FAS | rs2229521 | yes | 10 | 70000000 | 0.01096 |
|  | FAS | rs2234978 | yes | 10 | 90761809 | 0.004154 |
|  | FAS | rs28362318 | yes | 10 | 90758660 | 0.129167 |
|  | FAS | rs28362322 | yes | 10 | 90760534 | 0.173404 |
|  | FAS | rs3218611 | yes | 10 | 90764093 | 0.011375 |
|  | FAS | rs3218619 | yes | 10 | 90752781 | 0.332109 |
|  | FAS | rs3218621 | yes | 10 | 90752876 | 0.044792 |
|  | FASLG | rs10458360 | yes | 1 | 170900598 | 0.494223 |
|  | FASLG | rs16844869 | yes | 1 | 170899374 | 0.021807 |
|  | FASLG | rs17370527 | yes | 1 | 170899580 | 0.107781 |
|  | FASLG | rs2022556 | yes | 1 | 170897077 | 0.458774 |
|  | FASLG | rs5030772 | yes | 1 | 170899973 | 0.105538 |
|  | FASLG | rs6700734 | yes | 1 | 170897434 | 0.235511 |
|  | FASLG | rs929087 | yes | 1 | 170898680 | 0.414391 |
