# Supplementary material for: Identifying Host Genetic Risk Factors in the Context of Public Health Surveillance for Invasive Pneumococcal Disease
Source: PLoS One. 2011 Aug 15;6(8):e23413. doi: 10.1371/journal.pone.0023413 (PMC3156135; doi:10.1371/journal.pone.0023413)
Supplement: Table S2 — tagSNPs in candidate genes found associated with IPD. For European-Americans (182 Cases and 361 Controls), and African-Americans (53 Cases and 113 Controls). Allelic (2×2) and genotypic (2×3) models are used to calculate allelic, heterozygote and homozygote OR, 95% confidence intervals (CI) and minor allele frequency (MAF). Variants are ordered by decreasing significance of the allelic p-value. (DOC) [file pone.0023413.s002.doc]

**Table S2: tagSNPs in candidate genes found associated with IPD**

| **Gene** | **SNP** | **Allelic**  **p-value** | **Genotypic p-value** | **CodedAllele** | **Minor Allele** | **Heterozygote OR (95% CI)** | **Homozygote**  **OR (95% CI)** | **Allelic**  **OR (95% CI)** | **Control MAF** | **Case MAF** |
| --- | --- | --- | --- | --- | --- | --- | --- | --- | --- | --- |
| **European-Americans** | | |  |  |  |  |  |  |  |  |
| *IL10* | rs1800894 | 0.0038 | -- | A | A | 2.99 (1.39, 6.40) | -- | 2.89 (1.37, 6.12) | 0.02 | 0.05 |
| *SFTPD* | rs17886286 | 0.0073 | 0.0264 | G | C | 0.42 (0.22, 0.82) | 0.46 (0.05, 4.15) | 0.45 (0.25, 0.82) | 0.08 | 0.04 |
| *IL12B* | rs919766 | 0.0076 | 0.0396 | C | C | 1.77 (1.05, 2.97) | 2.91 (0.64, 13.16) | 1.85 (1.17, 2.92) | 0.06 | 0.11 |
| *SFTPA1* | rs4253457 | 0.0159 | 0.0372 | A | G | 0.46 (0.25, 0.84) | 0.73 (0.14, 3.80) | 0.52 (0.30, 0.89) | 0.09 | 0.05 |
| *IL12B* | rs2195940 | 0.0220 | 0.0503 | T | T | 1.53 (0.93, 2.51) | 6.45 (0.67, 62.56) | 1.68 (1.07, 2.63) | 0.07 | 0.11 |
| *SFTPD* | rs1998374 | 0.0233 | 0.0382 | A | G | 0.52 (0.31, 0.86) | 0.86 (0.21, 3.49) | 0.60 (0.39, 0.94) | 0.13 | 0.08 |
| *IL4* | rs2243302 | 0.0235 | 0.0487 | A | A | 1.76 (1.12, 2.78) | 1.11 (0.10, 12.37) | 1.61 (1.06, 2.45) | 0.08 | 0.13 |
| *SFTPA1* | rs1914663 | 0.0239 | 0.0664 | C | T | 0.50 (0.27, 0.91) | 0.62 (0.06, 6.01) | 0.53 (0.31, 0.93) | 0.09 | 0.05 |
| *CD46* | rs2488255 | 0.0277 | 0.0898 | C | C | 1.23 (0.80, 1.88) | 1.75 (1.06, 2.89) | 1.33 (1.03, 1.72) | 0.42 | 0.49 |
| *IL1B* | rs3917365 | 0.0313 | 0.0401 | T | T | 1.89 (1.23, 3.19) | -- | 1.71 (1.05, 2.81) | 0.05 | 0.09 |
| *IL1R1* | rs3917318 | 0.0376 | 0.0896 | A | G | 0.67 (0.46, 0.99) | 0.62 (0.29, 1.34) | 0.73 (0.54, 0.98) | 0.29 | 0.23 |
| *IL1R1* | rs2287047 | 0.0382 | 0.0566 | C | T | 0.63 (0.43, 0.92) | 0.72 (0.30, 1.72) | 0.72 (0.53, 0.98) | 0.26 | 0.20 |
| *IL1R1* | rs2160227 | 0.0403 | 0.1010 | C | A | 0.69 (0.47, 1.00) | 0.62 90.29, 1.32) | 0.74 (0.55, 0.99) | 0.28 | 0.23 |
| *IL1B* | rs2853550 | 0.0446 | 0.0276 | T | T | 1.90 (1.34, 3.18) | -- | 1.63 (1.01, 2.65) | 0.06 | 0.09 |
| *CD46* | rs1962149 | 0.0449 | 0.1343 | T | T | 1.25 (0.82, 1.91) | 1.69 (1.01, 2.82) | 1.30 (1.01, 1.68) | 0.42 | 0.48 |
| *FAS* | rs2234978 | 0.0454 | 0.1362 | C | T | 0.76 (0.52, 1.12) | 0.49 (0.20, 1.16) | 0.73 (0.54, 0.99) | 0.26 | 0.20 |
| *CD46* | rs2724385 | 0.0484 | 0.1228 | A | T | 1.48 (0.94, 2.33) | 1.66 (0.99, 2.77) | 1.30 (1.00, 1.68) | 0.47 | 0.53 |
| **African-Americans** | | |  |  |  |  |  |  |  |  |
| *PTAFR* | rs905907 | 0.0046 | 0.0071 | G | G | 3.21 (1.52, 6.76) | 1.93 (0.31, 12.13) | 2.38 (1.29, 4.39) | 0.11 | 0.24 |
| *SFTPD* | rs12219080 | 0.0091 | 0.0363 | C | T | 0.34 (0.13, 0.90) | -- | 0.32 (0.13, 0.78) | 0.16 | 0.06 |
| *IL1R1* | rs997049 | 0.0195 | 0.0613 | T | T | 2.40 (1.15, 5.19) | 2.14 (0.54, 8.50) | 2.02 (1.11, 3.67) | 0.14 | 0.24 |
| *SFTPD* | rs17878441 | 0.0205 | 0.0650 | G | A | 0.37 (0.14, 0.95) | -- | 0.36 (0.14, 0.88) | 0.15 | 0.06 |
| *IL1R1* | rs3917272 | 0.0209 | -- | C | C | 9.06 (0.99, 83.17) | -- | 8.75 (0.97, 79.22) | 0.00 | 0.04 |
| *IL1R1* | rs949963 | 0.0344 | 0.0979 | G | A | 0.55 (0.27, 1.12) | 0.31 (0.08, 1.19) | 0.57 (0.34, 0.96) | 0.38 | 0.25 |
| *IL1B* | rs1143642 | 0.0415 | 0.1573 | C | T | 0.59 (0.27, 1.30) | 0.22 (0.03, 1.79) | 0.51 (0.26, 0.98) | 0.22 | 0.13 |
| *CD46* | rs41317049_rsNA | 0.0418 | -- | C | C | 2.62 (1.05, 6.52) | -- | 2.42 (1.01, 5.78) | 0.05 | 0.11 |
| *IL1R1* | rs2287049 | 0.0425 | 0.1292 | T | T | 2.00 (0.92, 4.38) | 2.26 (0.89, 5.74) | 1.65 (1.02, 2.67) | 0.36 | 0.48 |
| *SFTPB* | rs3024798 | 0.0476 | 0.1224 | A | A | 1.97 (0.98, 3.99) | 2.72 (0.37, 20.24) | 1.79 (1.00, 3.21) | 0.15 | 0.24 |
