# Supplementary material for: Identifying Host Genetic Risk Factors in the Context of Public Health Surveillance for Invasive Pneumococcal Disease
Source: PLoS One. 2011 Aug 15;6(8):e23413. doi: 10.1371/journal.pone.0023413 (PMC3156135; doi:10.1371/journal.pone.0023413)
Supplement: Table S3 — tagSNPs in candidate genes found associated with bacteremia. For European-Americans (120 Cases and 361 Controls) with 213 SNPs passing QC (HWE >0.0001, MAF >0.01, Genotyping efficiency >90%) and African-Americans (39 Cases and 113 Controls) with 281 SNPs passing QC. Allelic (2×2) and genotypic (2×3) models are used to calculate allelic, heterozygote and homozygote OR, 95% confidence intervals (CI) and minor allele frequency (MAF). Variants are ordered by decreasing significance of the allelic p-value. (DOC) [file pone.0023413.s003.doc]

**Table S3: tagSNPs in candidate genes found associated with bacteremia**

| **Gene** | **SNP** | **Allelic**  **p-value** | **Genotypic p-value** | **Coded Allele** | **Minor Allele** | **Heterozygote**  **OR (95% CI)** | **Homozygote**  **OR (95% CI)** | **Allelic**  **OR (95% CI)** | **Control MAF** | **Case MAF** |
| --- | --- | --- | --- | --- | --- | --- | --- | --- | --- | --- |
| **European-Americans** | | |  |  |  |  |  |  |  |  |
| *SFTPA1* | rs4253457 | 0.0037 | 0.0190 | A | G | 0.39 (0.18, 0.84) | -- | 0.35 (0.16, 0.73) | 0.09 | 0.03 |
| *SFTPD* | rs17886286 | 0.0055 | 0.0253 | G | C | 0.37 (0.16, 0.84) | -- | 0.34 (0.15, 0.75) | 0.08 | 0.03 |
| *SFTPD* | rs17886630 | 0.0061 | 0.0288 | T | A | 0.52 (0.27, 0.97) | -- | 0.44 (0.24, 0.80) | 0.12 | 0.06 |
| *IL12B* | rs919766 | 0.0064 | 0.0347 | C | C | 1.90 (1.06, 3.40) | 3.34 (0.66, 16.83) | 2.00 (1.21, 3.31) | 0.06 | 0.11 |
| *SFTPD* | rs1998374 | 0.0121 | 0.0410 | A | G | 0.54 (0.30, 0.97) | -- | 0.50 (0.29, 0.87) | 0.13 | 0.07 |
| *IL12A* | rs2243148 | 0.0126 | 0.0169 | A | G | 0.53 (0.33, 0.83) | 0.61 (0.27, 1.32) | 0.65 (0.46, 0.91) | 0.31 | 0.23 |
| *CD46* | rs7545126 | 0.0214 | 0.0605 | C | T | 0.57 (0.33, 1.00) | -- | 0.54 (0.32, 0.92) | 0.13 | 0.08 |
| *IL18* | rs5744247 | 0.0215 | 0.0853 | C | G | 0.53 (0.28, 1.00) | 0.34 (0.04, 2.72) | 0.52 (0.29, 0.92) | 0.11 | 0.06 |
| *IL12B* | rs2195940 | 0.0267 | 0.0586 | T | T | 1.62 (0.92, 2.83) | 6.61 (0.59, 73.70) | 1.76 (1.06, 2.91) | 0.07 | 0.11 |
| *IL1R1* | rs2228139 | 0.0314 | 0.0261 | G | G | 1.98 (1.69, 3.37) | -- | 1.71 (1.04, 2.80) | 0.07 | 0.11 |
| *SFTPD* | rs2181204 | 0.0323 | 0.0850 | T | C | 0.64 (0.37, 1.11) | -- | 0.58 (0.34, 0.96) | 0.13 | 0.08 |
| *CD46* | rs41317049_rsNA | 0.0368 | 0.0886 | A | C | 0.64 (0.38, 1.09) | -- | 0.59 (0.36, 0.97) | 0.14 | 0.09 |
| *IL1R1* | rs3917332 | 0.0372 | 0.1007 | A | A | 1.56 (1.00, 4.33) | 1.78 (0.64, 4.94) | 1.45 (1.02, 2.07) | 0.18 | 0.25 |
| *IL1R1* | rs3917289 | 0.0414 | 0.0351 | A | T | 1.94 (1.14, 3.32) | -- | 1.68 (1.02, 2.76) | 0.07 | 0.11 |
| *IL4* | rs2243302 | 0.0422 | 0.1096 | A | A | 1.73 (1.03, 2.92) | 1.70 (0.15, 19.98) | 1.63 (1.01, 2.62) | 0.08 | 0.13 |
| *IL18* | rs5744258 | 0.0431 | 0.1322 | C | C | 1.35 (0.87, 2.10) | 2.04 (0.92, 4.50) | 1.41 (1.01, 1.96) | 0.22 | 0.29 |
| *MYD88* | rs7744 | 0.0458 | 0.0122 | G | G | 1.95 (1.23, 3.10) | 0.67 (0.15, 3.11) | 1.49 (1.01, 2.20) | 0.14 | 0.19 |
| **African-Americans** | | |  |  |  |  |  |  |  |  |
| *IL1B* | rs1143642 | 0.0056 | 0.0348 | C | T | 0.40 (0.15, 1.06) | -- | 0.30 (0.12, 0.74) | 0.22 | 0.08 |
| *IL1R1* | rs2287049 | 0.0135 | 0.0321 | T | T | 2.90 (1.15, 7.29) | 3.29 (1.13, 9.58) | 1.99 (1.17, 3.39) | 0.36 | 0.53 |
| *IL1R1* | rs949963 | 0.0149 | 0.0368 | G | A | 0.40 (0.18, 0.91) | 0.25 (0.05, 1.22) | 0.46 (0.258, 0.85) | 0.38 | 0.22 |
| *IL4* | rs2243219 | 0.0212 | 0.0676 | G | G | 2.34 (0.88, 6.18) | 3.27 (1.12, 9.53) | 1.89 (1.11, 3.23) | 0.41 | 0.57 |
| *IL1B* | rs2853550 | 0.0214 | 0.0768 | C | T | 0.58 (0.26, 1.25) | 0.15 (0.02, 1.19) | 0.48 (0.26, 0.90) | 0.33 | 0.19 |
| *IL1R1* | rs997049 | 0.0301 | 0.0592 | T | T | 2.58 (1.11, 6.03) | 2.26 (0.51, 10.21) | 2.13 (1.11, 4.07) | 0.14 | 0.25 |
| *IL1B* | rs3917365 | 0.0347 | 0.1099 | C | T | 0.38 (0.14, 1.06) | 0.32 (0.04, 2.74) | 0.40 (0.17, 0.94) | 0.20 | 0.09 |
| *PTAFR* | rs905907 | 0.0362 | 0.0206 | G | G | 2.99 (1.32, 6.77) | 1.26 (0.13, 12.69) | 2.15 (1.09, 4.25) | 0.11 | 0.22 |
| *MBL2* | rs930507 | 0.0481 | 0.0410 | G | G | 2.80 (1.22, 6.45) | 2.07 (0.56, 7.69) | 1.82 (1.03, 3.21) | 0.26 | 0.39 |
| *IL1R1* | rs2287047 | 0.0482 | 0.0882 | C | C | 2.24 (0.77, 6.53) | 3.50 (1.09, 11.22) | 1.75 (1.04, 2.95) | 0.46 | 0.60 |
| *SFTPD* | rs12219080 | 0.0494 | 0.1343 | C | T | 0.40 (0.14, 1.13) | -- | 0.36 (0.14, 0.97) | 0.16 | 0.07 |
