# Supplementary material for: Identifying Host Genetic Risk Factors in the Context of Public Health Surveillance for Invasive Pneumococcal Disease
Source: PLoS One. 2011 Aug 15;6(8):e23413. doi: 10.1371/journal.pone.0023413 (PMC3156135; doi:10.1371/journal.pone.0023413)
Supplement: Table S4 — tagSNPs in candidate genes associated with pneumonia and meningitis for European-Americans (EA). 25 Cases and 361 Controls with 211 SNPs passing QC (HWE >0.0001, MAF >0.01, Genotyping efficiency >90%). Allelic (2×2) and genotypic (2×3) models are used to calculate allelic, heterozygote and homozygote OR, 95% confidence intervals (CI) and minor allele frequency (MAF). Variants are ordered by decreasing significance of the allelic p-value. (DOC) [file pone.0023413.s004.doc]

**Table S4: tagSNPs in candidate genes associated with pneumonia and meningitis for European-Americans (EA)**

| **Gene** | **SNP** | **Allelic**  **p-value** | **Genotypic p-value** | **Coded Allele** | **Minor Allele** | **Heterozygote**  **OR (95% CI)*** | **Homozygote**  **OR (95% CI)*** | **Allelic**  **OR (95% CI)*** | **Control MAF** | **Case MAF** |
| --- | --- | --- | --- | --- | --- | --- | --- | --- | --- | --- |
| **Pneumococcal Pneumonia - EA** | | |  |  |  |  |  |  |  |  |
| *SFTPD* | rs17886233 | *0.0017* | 0.0022 | T | T | 1.05 (0.02, 11.5) | -- | 6.04 (2.27, 16.1) | 0.03 | 0.16 |
| *IL10* | rs1800894 | *0.0021* | 0.0017 | A | A | 3.19 (0.00, 29.2) | -- | 7.37 (2.47, 22.0) | 0.02 | 0.12 |
| *SFTPD* | rs17886252 | *0.0033* | 0.0033 | T | T | 1.49 (0.12, 13.8) | 5.98 (0.00, 70.4) | 4.47 (1.83, 10.9) | 0.05 | 0.18 |
| *SFTPD* | rs17886630 | *0.0057* | 0.0179 | A | A | 0.61 (0.05, 4.52) | 3.51, (0.00, 38.5) | 2.96 (1.42, 6.15) | 0.11 | 0.26 |
| *IL12A* | rs2243135 | *0.0225* | 0.0655 | C | C | 2.17 (0.20, 111.4) | 1.79 (0.02, 147.1) | 2.10 (1.12, 3.95) | 0.39 | 0.57 |
| *IL12A* | rs2243149 | *0.0341* | 0.0568 | A | A | 1.05 (0.09, 54.9) | 0.73 (0.01, 60.6) | 2.03 (1.08, 3.82) | 0.40 | 0.57 |
| *SFTPD* | rs7078012 | *0.0404* | 0.0289 | T | T | 1.43 (0.10, 20.5) | 3.23 (0.05, 69.0) | 2.33 (1.07, 5.07) | 0.10 | 0.21 |
| *CTLA4* | rs231775 | *0.0448* | 0.1103 | G | G | 0.16 (0.00, 1.52) | 0.35 (0.00, 2.77) | 1.96 (1.03, 3.74) | 0.38 | 0.55 |
| *CTLA4* | rs231779 | *0.0496* | 0.1205 | T | T | 0.32 (0.03, 2.37) | 0.27 (0.00, 2.29) | 1.93 (1.03, 3.62) | 0.39 | 0.55 |
| *SERPINE1* | rs2227631 | *0.5188* | 0.0207 | A | G | 0.51 (0.02, 31.9) | 1.03 (0.07, 58.4) | 0.77 (0.40, 1.47) | 0.42 | 0.36 |
| *IL18* | rs795467 | *0.3749* | 0.0251 | A | A | 0.42 (0.01, 3.95) | 2.59 (0.00, 24.6) | 1.35 (0.70, 2.63) | 0.27 | 0.33 |
| *IL1R1* | rs3917254 | *0.7738* | 0.0436 | A | A | 0.63 (0.01, 8.19) | 5.49 (0.40, 57.4) | 1.15 (0.39, 3.33) | 0.08 | 0.10 |
| **Pneumococcal Meningitis - EA** | | |  |  |  |  |  |  |  |  |
| *LY96* | rs6472812 | 0.001 | 0.003 | A | A | 5.67 (1.20, 21.3) | 27.3 (0.33, 2239.5) | 6.78 (2.53, 18.1) | 0.03 | 0.19 |
| *IL1B* | rs3917366 | 0.016 | 0.033 | A | A | 2.94 (0.86, 11.4) | 4.70 (0.69, 25.9) | 2.39 (1.19, 4.81) | 0.25 | 0.44 |
| *IL1B* | rs1143637 | 0.016 | 0.037 | A | A | 3.16 (0.92, 12.3) | 4.22 (0.62, 23.2) | 2.39 (1.19, 4.81) | 0.25 | 0.44 |
| *IL6* | rs2069845 | 0.018 | 0.054 | A | G | 0.46 (0.14, 1.52) | 0.12 (0.00, 0.96) | 0.39 (0.18, 0.85) | 0.50 | 0.28 |
| *SFTPD* | rs721917 | 0.020 | 0.049 | T | C | 0.50 (0.14, 1.72) | 0.13 (0.00, 0.90) | 0.37 (0.15, 0.88) | 0.48 | 0.25 |
| *IL1B* | rs1143634 | 0.021 | 0.040 | T | T | 2.86 (0.80, 11.4) | 4.39 (0.64, 24.1) | 2.40 (1.17, 4.94) | 0.24 | 0.44 |
| *IL10* | rs1878672 | 0.028 | 0.074 | G | G | 2.28 (0.42, 22.9) | 5.42 (0.99, 55.0) | 2.40 (1.14, 5.06) | 0.44 | 0.66 |
| *SFTPD* | rs1923537 | 0.029 | 0.077 | A | G | 0.35 (0.09, 1.12) | 0.21 (0.00, 1.49) | 0.39 (0.17, 0.91) | 0.40 | 0.21 |
| *MBL2* | rs2099903 | 0.029 | 0.037 | A | A | 3.28 (0.96, 12.8) | 4.19 (0.37, 27.8) | 2.37 (1.14, 4.91) | 0.22 | 0.41 |
| *SFTPD* | rs2245545 | 0.030 | 0.071 | A | G | 1.12 (0.21, 7.36) | 3.70 (0.85, 22.3) | 2.27 (1.08, 4.79) | 0.46 | 0.66 |
| *SFTPD* | rs2758554 | 0.031 | 0.143 | C | T | 0.39 (0.09, 1.31) | 0.30 (0.00, 1.88) | 0.32 (0.11, 0.92) | 0.29 | 0.12 |
| *MBL2* | rs2165813 | 0.032 | 0.043 | A | A | 3.23 (0.94, 12.6) | 3.77 (0.34, 24.9) | 2.29 (1.10, 4.73) | 0.23 | 0.41 |
| *CRP* | rs1417938 | 0.040 | 0.068 | A | A | 1.53 (0.41, 6.26) | 4.18 (0.91, 19.2) | 2.10 (1.05, 4.19) | 0.32 | 0.50 |
| *SFTPD* | rs1923541 | 0.040 | 0.050 | C | C | 0.82 (0.13, 5.70) | 3.32 (0.77, 20.2) | 2.25 (1.04, 4.89) | 0.47 | 0.67 |
| *IL1B* | rs3136558 | 0.041 | 0.084 | C | C | 2.29 (0.68, 8.23) | 3.83 (0.58, 19.3) | 2.15 (1.06, 4.36) | 0.25 | 0.41 |
| *SFTPD* | rs1570328 | 0.044 | 0.013 | C | C | 0.42 (0.03, 3.72) | 3.06 (0.70, 18.5) | 2.43 (1.04, 5.67) | 0.48 | 0.69 |
| *IL12B* | rs2569253 | 0.044 | 0.072 | C | T | 1.22 (0.19, 13.1) | 3.74 (0.74, 36.6) | 0.43 (0.20, 0.94) | 0.48 | 0.28 |
| *SFTPD* | rs2758545 | 0.044 | 0.082 | T | T | 1.63 (0.40, 7.79) | 4.00 (0.90, 20.1) | 2.14 (1.07, 4.30) | 0.37 | 0.56 |
| *SFTPD* | rs2819097 | 0.045 | 0.095 | G | G | 1.64 (0.41, 7.85) | 3.91 (0.89, 19.7) | 2.13 (1.06, 4.27) | 0.37 | 0.56 |
| *IL1R1* | rs3917289 | 0.045 | 0.052 | T | T | 3.40 (0.98, 10.6) | 10.8 (0.00, 142.8) | 2.66 (1.05, 6.74) | 0.07 | 0.18 |
| *IL1B* | rs3917368 | 0.046 | 0.150 | C | T | 0.39 (0.10, 1.26) | 0.25 (0.01, 1.79) | 0.43 (0.18, 1.00) | 0.38 | 0.21 |
